# Effects of Prenatal Essential and Toxic Metal Exposure on Children’s Neurodevelopment: A Multi-Method Approach

**DOI:** 10.3390/toxics13110954

**Published:** 2025-11-05

**Authors:** Xiruo Kou, Stefano Renzetti, Josefa Canals, Stefano Calza, Cristina Jardí, Victoria Arija

**Affiliations:** 1 Nutrition and Mental Health (NUTRISAM) Research Group, Universitat Rovira i Virgili, 43204 Reus, Spaincristina.jardi@urv.cat (C.J.); 2 Institut d’Investigació Sanitaria Pere Virgili (IISPV), 43204 Reus, Spain; 3 Department of Medical and Surgical Specialties, Radiological Sciences, and Public Health, Università Degli Studi di Brescia, 43201 Brescia, Italy; 4 Centre de Recerca en Avaluació i Mesura de la Conducta (CRAMC), Department of Psychology, Universitat Rovira i Virgili, 43007 Tarragona, Spain; 5 University Research Institute on Sustainability, Climate Change and Energy Transition (IU-RESCAT) Universitat Rovira i Virgili, 43003 Tarragona, Spain; 6 Department of Molecular and Translational Medicine, Università Degli Studi di Brescia, 43201 Brescia, Italy; 7 Collaborative Research Group on Lifestyles, Nutrition and Smoking (CENIT), Tarragona-Reus Research Support Unit, Jordi Gol Primary Care Research Institute (IDIAP Jordi Gol), 43003 Tarragona, Spain

**Keywords:** prenatal metal exposure, neurodevelopment, essential metals, toxic metals, weighted quantile sum regression

## Abstract

The impact of prenatal exposure to trace metal mixtures on children’s neurodevelopment remains debated. Many studies treat all trace metals as a single entity, overlooking the distinct biological roles of essential and toxic metals. This approach may highlight overall exposure but fails to capture their differential effects on neurodevelopment. This study aims to examine the associations between prenatal exposure to essential and toxic metals and children’s cognitive development, focusing on their independent effects. A cohort of 201 mother–infant pairs was analyzed. Maternal urinary metal levels were measured at 12 weeks of gestation, and children’s neurodevelopment was assessed at 4 years using the Wechsler Preschool and Primary Scale of Intelligence and the Developmental Neuropsychological Assessment. Generalized Additive Models (GAM), Restricted Cubic Spline (RCS), and Weighted Quantile Sum (WQS) regression were applied. GAM identified non-linear associations between essential metals (manganese and molybdenum) and cognitive outcomes, including verbal comprehension index (VCI), working memory index, full-scale IQ, and general ability index, which were confirmed by RCS. No non-linear relationships were observed for toxic metals. WQS showed negative associations between toxic metals and VCI (b = −1.07), processing speed index (b = −0.98), vocabulary acquisition index (b = −1.25), and verbal fluency (b = −0.23), mainly driven by cadmium (Cd) and antimony (Sb). Essential metal mixtures were not associated with cognitive outcomes. Prenatal exposure to toxic metals negatively affects children’s cognitive and neuropsychological development. Reducing maternal exposure during pregnancy is essential for protecting offspring development.

## 1. Introduction

Early childhood is viewed as a sensitive period of neurocognitive development characterized by rapid brain growth and the formation of numerous neural connections [1]. During this period, the development of cognitive, language, and socio-emotional abilities, along with the acquisition of motor and perceptual skills, lays the foundation for formal learning processes [2]. Cognitive development is characterized by the emergence of executive functions, including attention, working memory, and inhibitory control, as well as symbolic thinking and problem-solving skills. Early cognitive difficulties may have a lasting negative impact on cognitive abilities, as well as on adaptive and functional capacities [3,4]. Therefore, understanding the factors that lead to early cognitive difficulties is crucial for promoting children’s healthy development and ensuring reach their full potential.

Metals can enter the body through various pathways, including diet, water, inhalation, and other environmental sources, and their accumulation may result in neurotoxicity [5]. Prenatal exposure to metals is particularly concerning, as certain metals can cross the placental barrier and interfere with fetal brain development [6]. Essential metals play a vital role in neurobiological processes, and while metals such as manganese (Mn) are particularly important for neurodevelopment, they can have detrimental effects on cognitive function if present in either insufficient or excessive amounts [7]. In contrast, toxic metals like arsenic (As), lead (Pb), and cadmium (Cd) are well-established neurotoxicants with harmful effects, even at low levels [8].

Recent advances in statistical methods have allowed for the exploration of the effect of metal mixtures on neurodevelopment [9,10]. However, findings remain inconsistent. Some studies suggest that prenatal exposure to metal mixtures may impair various cognitive and developmental functions, such as learning abilities [11], reward motivation [12], visuospatial skills [13], visual processing [14], temporal processing [15], language and motor development [16], and even children’s intelligence quotient (IQ) [17,18,19,20]. Additionally, prenatal multiple metals exposure may contribute to the risk of developing neurodevelopmental disorders, such as autism or attention deficit hyperactivity disorder (ADHD) [21,22]. However, other studies have found no significant link between metal mixtures and these conditions [23,24]. Previous studies often combine essential and toxic metals into a single mixture, potentially obscuring their independent effects [25]. Separating essential and toxic metals enables for a clearer understanding of how each group independently influences cognitive outcomes, as the harmful and protective effects of these metals may differ notably within their respective groups [26,27]. Given the distinct biological mechanisms of each group, this separation is essential for understanding their unique contributions to neurodevelopment.

Therefore, the current analysis aims to explore the effects of prenatal exposure to eight essential metals and six toxic metals on cognitive development in children, with a focus on distinguishing the independent effects of these two groups to better inform public health interventions.

## 2. Methods

### 2.1. Study Design

The analysis was conducted on a subgroup of participants from the ECLIPSES Study, a community-based randomized controlled trial focusing on iron supplementation among pregnant women in Tarragona, Catalonia, Spain. The study was designed in accordance with the Declaration of Helsinki and was approved by the Ethics Committee of the Institut d’Investigació en Atenció Primaria de Salut (IDIAP) and the Institut d’Investigació Sanitària Pere Virgili (approval ID: 118/2017; date: 28 September 2017). Informed consent forms were signed by all women who participated. Participants were recruited from primary care centers during their initial prenatal visits with midwives between 2013 and 2017. Detailed inclusion and exclusion criteria are available in a previous publication [28]. Out of the 791 women initially recruited, 257 were lost to follow-up due to the following reasons: voluntary withdrawal (*n* = 180), miscarriage (*n* = 13), meeting exclusion criteria (*n* = 46), and unknown reasons (*n* = 18). This left 534 women who reached the third trimester of pregnancy and completed the study. Among their children, psychological data at 4 years of age were available for 231 cases. However, prenatal maternal urine samples and metal analysis were not available for 30 cases. As a result, the final analysis included 201 mother–infant pairs with both prenatal urine samples and neurodevelopmental data available.

### 2.2. Maternal Baseline Data Information

Maternal baseline information was collected through structured interviews conducted at enrollment. These interviews covered demographic details, body mass index (BMI, kg/m^2^), socioeconomic status (based on educational level and occupation), smoking habits (categorized as never smoked, former smoker, or current smoker), and adherence to the Mediterranean diet [29]. Psychological distress was assessed in both the first and third trimesters using the State-Trait Anxiety Inventory, and the average of these two scores represented overall distress during pregnancy [30].

### 2.3. Urinary Sample Collection and Metal Analysis

During the first trimester, at the 12th week of gestation, maternal urine samples were collected from the participants, homogenized, and then portioned for storage. These samples were cryopreserved in polypropylene vials at −20 °C for the subsequent analysis of trace elements including Magnesium (Mg), Chromium (Cr), Mn, Molybdenum (Mo), Cobalt (Co), Copper (Cu), Zinc (Zn), Selenium (Se), As, Cd, Antimony (Sb), Mercury (Hg), Pb, Nickel (Ni). Prior to the analytical procedures, the samples were allowed to equilibrate to room temperature and were diluted at a ratio of 1:10 with a 2% nitric acid solution, aligning with the diluent used for the calibration standards. For internal quality control, Lyphochek Urine Metal Controls for trace elements, Levels I and II (Bio-rad), were used, with observed values correlating with the certified values. The quantification of trace elements in the urine was performed using a Triple Quadrupole Inductively Coupled Plasma Mass Spectrometer (ICP-MS/MS) (Agilent 8800 triple quadrupole ICP-MS from Agilent Technologies, Santa Clara, CA, USA). The methodology for the determination of these metals has been detailed somewhere else [31,32]. Blank samples for our analysis were prepared with a 2% nitric acid solution. The limits of detection (LOD) in units of µg/L for each metal were as follows: Mg (2), Cr (0.05), Mn (0.04), Co (0.004), Ni (0.2), Cu (0.1), Zn (1), As (0.1), Se (1), Mo (0.02), Cd (0.02), Sb (0.03), Hg (0.03), and Pb (0.07). To account for variations in urine concentration, the levels of trace metals in the urine samples were normalized to their respective creatinine levels. Creatinine levels in the urine were quantified using the ADVIA 1800 and 2400 Chemistry Systems [33]. For standardization and to adjust for differences in urine dilution, the concentrations of metals in the urine were expressed in micrograms per gram of creatinine [32].

### 2.4. Children Neurodevelopment

Two trained psychologists carried out individualized cognitive assessments for the children at 4 years of age. These evaluations were performed using the Spanish version of the Wechsler Preschool and Primary Scale of Intelligence (WPPSI-IV) [34] and the Developmental Neuropsychological Assessment (NEPSY-II) [35]. WPPSI-IV evaluates cognitive abilities through 15 sub-tests. Composite scores were calculated based on primary indexes, including the Verbal Comprehension Index (VCI), Fluid Reasoning Index (FRI), Working Memory Index (WMI), Processing Speed Index (PSI), and Full-Scale IQ (FSIQ). Additional secondary indexes included the Vocabulary Acquisition Index (VAI), Nonverbal Index (NVI), and General Ability Index (GAI). All indexes have a mean of 100 and a standard deviation of 15.

The NEPSY-II is a versatile neuropsychological test battery aimed at evaluating neurocognitive abilities. In this study, only sub-tests that complemented the skills assessed by the WPPSI-IV were utilized: verbal fluency (language domain), visual-motor precision (sensorimotor domain) and emotion recognition (social perception domain). The sub-tests of the NEPSY--II have a mean of 10 and an SD of 3.

### 2.5. Statistical Analysis

The population characteristics were summarized with categorical variables presented as frequencies and percentages, while continuous variables were reported as means with standard deviations (SD). All metal exposures were transformed into deciles, applying thresholds based on the decile distribution of each metal’s concentration to standardize measurements.

The analysis proceeded as follows: First. Spearman correlation coefficients were estimated between prenatal urine metal concentrations, which provided insight into potential multicollinearity issues and relationships among the metals [36]. Second, Generalized Additive Models (GAMs) were employed to explore the relationships between individual metal exposures and neurodevelopmental outcomes, detecting both linear and non-linear patterns of association. The results of the GAMs analysis guided subsequent modeling steps [37]. Then, Restricted Cubic Splines (RCS) were applied as a secondary method to verify and further characterize the non-linear associations identified by GAMs, ensuring the robustness of the observed patterns and trends [38]. Third, Weighted Quantile Sum (WQS) regression models were fitted for each neurodevelopmental outcome [10]. WQS regression consists of two steps: a training step where the weights are estimated through a bootstrap to build the WQS index and a validation step where the index is included in the regression model as a covariate. In the present study, we applied a variation of the model where we considered two separate indices, one containing the essential metals and one the toxic metals. In the training step, the weights of the indices were estimated separately, while in the validation step, we included both indices in the same regression model. Both the positive and negative direction of the association between each index and each outcome were explored and the models with the lowest Akaike Information Criterion were selected. To capture potential non-linear associations, quadratic terms were added to the WQS models for outcomes where GAMs suggested curvilinear relationships. All metal exposures were transformed into deciles, which were then combined into a weighted index. Each WQS model was validated using a 60/40 training-validation split. To enhance reliability, 100 bootstrap samples were generated, and the training-validation steps were repeated 100 times, following a repeated holdout validation procedure [39]. A penalization term equal to 100 was applied to the estimate of the weights in the training step to obtain better parameter estimates [40]. Covariates in all analyses included maternal age, BMI, socioeconomic status, smoking status, Mediterranean diet adherence during pregnancy, child gender, and feeding methods. These variables were selected based on prior literature and their potential to confound the associations between prenatal metal exposure and neurodevelopmental outcomes. All statistical analyses were performed using R version 4.4.1. The *mgcv* package was used for GAMs [37], the *rms* package was utilized to implement RCS [41] and the *gWQS* package was utilized for WQS regression [42]. Statistical significance was set at α = 0.05, with all tests conducted as two-sided.

## 3. Results

### 3.1. Maternal and Offspring Characteristics and Neurodevelopmental Outcomes

The study population included 201 mother-offspring pairs. Mothers had an average age of 31.6 years and a mean BMI of 24.7. The majority were from low or medium social classes (74.6%), with 67.7% identified as never smokers. The average Mediterranean diet score during pregnancy was 9.65. Neurodevelopmental assessments showed average scores on the WPPSI, with the VCI being 105.27 (SD = 13.63), FRI 102.67 (SD = 12.81), and WMI 97.75 (SD = 12.18). All these scores fall within the typical range, suggesting normal cognitive abilities.

Additionally, scores on the NEPSY assessment showed that the average verbal fluency score was 9.02 (SD = 2.88), visual-motor precision was 10.32 (SD = 3.28), and emotion recognition was 9.01 (SD = 2.50). These scores, while variable, are generally consistent with the typical developmental range, suggesting that the child’s language, motor, and emotional recognition skills are within normal developmental expectations (Table 1).

### 3.2. Urinary Concentrations of Metals in Pregnant Women

Urinary concentrations of metals in pregnant women are summarized in Table 2. Urinary concentrations of metals in pregnant women are summarized in Table 2. Among essential metals, the geometric mean (GM) concentrations were highest for Mg (7.43 × 10^4^ μg/g creatinine) and Zn (271.80 μg/g creatinine), while the lowest was observed for Mn (0.11 μg/g creatinine). For toxic metals, As had the highest GM (30.44 μg/g creatinine), followed by Hg (0.50 μg/g creatinine) and Pb (0.37 μg/g creatinine).

**Figure 1:**
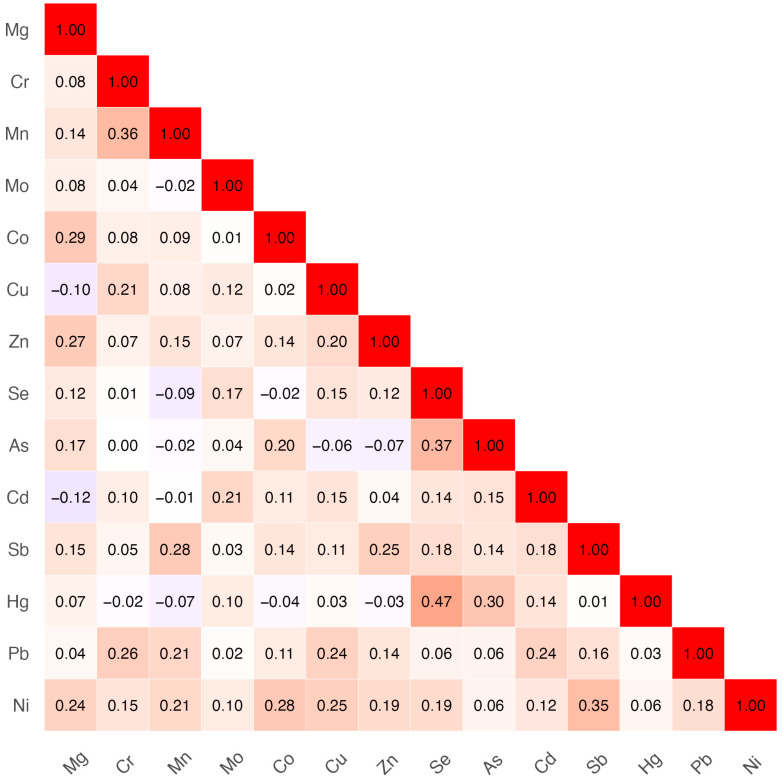
Spearman correlation among trace metals.

The overall levels of both essential and toxic metals were comparable to those reported in other European populations. Figure 1 indicated a lack of strong correlations among metals, suggesting that each exposure may contribute independently to potential neurodevelopmental effects. Some correlations were moderate (absolute value > 0.2), and the magnitude depended on the observed concentration ranges.

### 3.3. Correlation Between Metals and Neurodevelopmental Outcomes

**Figure 2:**
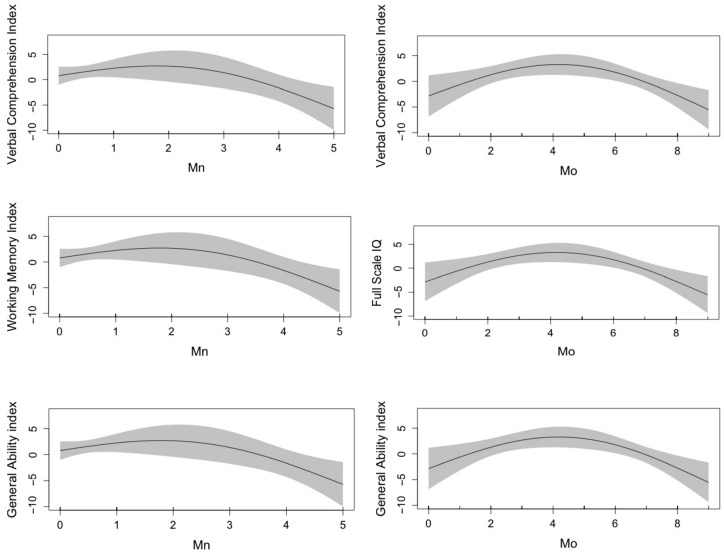
Plot of non-linear associations between metals and neurodevelopmental outcomes by Restricted Cubic Splines.

Supplementary Table S1 presented the effective degrees of freedom (edf) and significance of the smooth terms for metals and WPPSI scores obtained from GAMs. Non-linear relationships were observed for several metals with outcomes. Notably, Mn (edf = 1.81) and Mo (edf = 1.90) with VCI, Mn with WMI (edf = 1.87), Mo with FSIQ (edf = 1.87), and Mn (edf = 1.70) and Mo (edf = 1.91) with GAI. Additionally, RCS analysis confirmed the non-linear relationships between the metals and outcomes highlighted by GAM, as depicted in Figure 2. These results suggest that both low and high exposure levels of certain essential metals may be associated with less favorable cognitive outcomes. However, no significant non-linear relationships were observed between metals and NEPSY score (Supplementary Table S2).

### 3.4. The Joint Effect of Essential and Toxic Metals on Neurodevelopmental Outcomes

**Figure 3:**
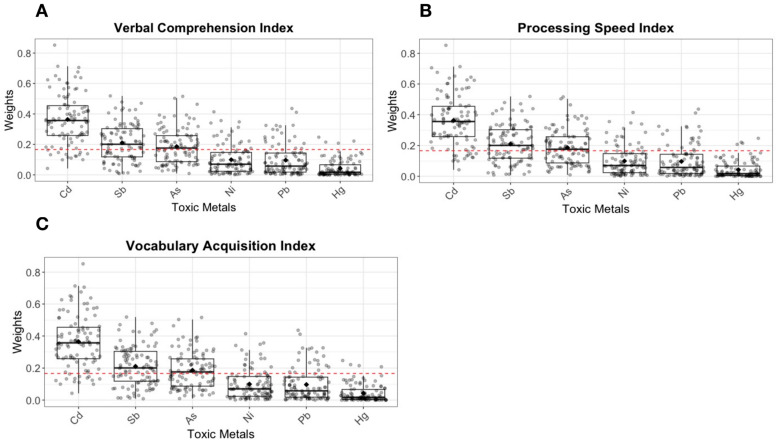
Weighted contributions of toxic metals to certain WPPSI scores by WQS. The contributions of toxic metals to VCI (**A**), PSI (**B**), and VAI (**C**). VCI, Verbal Comprehension Index; PSI, Processing Speed Index; VAI, Verbal Ability Index. The dotted red line represents the null contribution (zero effect) and serves as a reference for assessing the relative importance of each metal.

**Figure 4:**
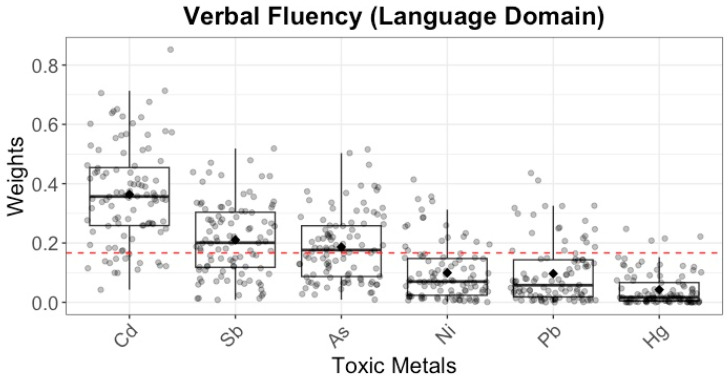
Weighted contributions of toxic metals to verbal fluency scores from NEPSY score by WQS. The dotted red line represents the null contribution (zero effect) and serves as a reference for assessing the relative importance of each metal.

Based on the GAM results, non-linear relationships were modeled as quadratic terms in the WQS analysis (Table 3). For WPPSI scores, the toxic metals mixture showed negative associations with VCI (b = −1.07), primarily driven by Cd (Figure 3A); with PSI (b = −0.98), with Sb playing a dominant role (Figure 3B); and with VAI (b = −1.25), again with Cd as the main contributor (Figure 3C). For NEPSY score, the toxic metals mixture was negatively associated with verbal fluency (language domain) (b = −0.23, 95% CI: −0.38, −0.03) (Table 4), with Cd and Sb as key contributors (Figure 4).

## 4. Discussion

In this prospective study, we found that prenatal exposure to toxic metal mixtures was associated with impaired neurodevelopment, particularly in test related to verbal and language areas, as indicated by decreased scores in VCI, VAI, and verbal fluency. In addition, prenatal exposure was negatively associated with efficiency and speed of cognitive processing (PSI). Among the toxic metals, Cd was the primary contributor to the language-related cognitive abilities, which has impact on verbal intelligence. While Sb predominantly drove the adverse effects on lower processing, which is crucial for learning efficiency. By contrast, the mixture of essential metals showed no significant association with neurodevelopmental outcomes. To the best of our knowledge, this is the first study to separately examine the prenatal effects of toxic and essential metal mixtures on children’s neurodevelopment, driven by the distinct biological mechanisms of these two groups. This approach offers novel insights into their differential contributions and underscores the need for targeted strategies to mitigate risks and promote healthy development.

In this study, we observed non-linear relationships between essential metals and certain neurodevelopmental outcomes. Among these, Mn and Mo exhibited significant inverted U-shaped associations. This finding aligns with previous research that reported an inverted U-shaped relationship between maternal Mn levels and children’s neurodevelopment [23,43,44,45]. Mn is essential for numerous biological functions; however, both deficiency and excessive levels may result in adverse neurological effects [27]. The concentration of Mn in our population (0.11 μg/g) is comparable to levels reported in America (0.18 μg/L) and France (<0.12 μg/L), but it is slightly lower than the levels observed in a similar region in Spain (0.38 μg/g) [46,47,48]. One possible explanation is that Mn deficiency, although rare, may disrupt neurotransmitter synthesis, mitochondrial function, and synaptic development. Conversely, excess Mn can induce oxidative stress, neuroinflammation, and disturbances in metal homeostasis, ultimately impairing neurodevelopment [49,50]. For Mo, human data on its dual effects on neurodevelopment is limited, but some evidence suggests that excessive Mo may disrupt copper metabolism and contribute to oxidative stress, potentially impairing neurodevelopment [51]. The level of Mo in the studied population (39.87 μg/g) is higher than that reported in a reasonably large industrial Spanish city (25.37 μg/g) but lower than levels observed in areas with similar living conditions in Spain (60.47 μg/g) [47]. Although primary Mo deficiency is rare, it may impair enzymes involved in sulfur metabolism and purine degradation, potentially leading to toxic effects [52]. However, no significant non-linear relationships were found between toxic metals exposure and neurodevelopmental outcomes. This may be because toxic metals typically have linear or threshold effects, where even low levels can disrupt neurodevelopment [8]. Unlike essential metals, toxic metals do not have beneficial roles in the body, so their effects are more directly harmful [5,8]. The non-linear associations observed for certain essential metals, such as Mn and Mo, are derived from population-level modeling using GAM. These models explore dose–response patterns across the observed exposure range, but it is important to note that individual exposure profiles vary and that a single measurement may not capture this variability fully. Thus, the curves should be interpreted as reflecting trends in the population rather than precise individual dose–response relationships.

In recent years, an increasing number of studies have examined the impact of prenatal polymetallic exposures on neurodevelopmental outcomes in offspring. However, the results have been inconsistent. Some studies have found no overall relationship between exposure to trace metals and neurodevelopmental indices, such as ADHD or cognitive outcomes [37,53]. In contrast, other research has shown that joint exposure to multiple trace metals is linked to poor neurodevelopmental outcomes, including motor problems, cognitive difficulties, or neurobehavioral issues [19,44,54]. A key limitation in many of these studies is treating all trace metals as a single homogeneous mixture, which overlooks the distinct biological mechanisms of essential and toxic metals. Essential metals are critical for normal biological processes, whereas toxic metals can disrupt cellular functions and impair health even at low concentrations [7,26]. Pooling all metals together may highlight overall exposure but fails to capture the differences in their impact on neurodevelopment [7,25]. In the current study, we addressed this limitation by using separate indices for toxic and essential metals, allowing for a more accurate assessment of each group’s contribution to neurodevelopment. Our findings showed that the mixture of essential metals was not related to neurodevelopmental outcomes, while the mixture of toxic metals had an adverse effect on VCI, PSI, VAI, and verbal fluency. Specifically, Cd was the primary contributor to deficits in VCI, VAI, and verbal fluency, which are strong predictors of later academic success, cognitive development, and social-emotional skills in early childhood. On the other hand, Sb predominantly drove the adverse effects on PSI and also contributed to impairments in verbal fluency. The negative impact of prenatal Cd exposure on children’s neurodevelopment is widely supported. A prospective study in Korea discovers that maternal blood Cd is adversely correlated with IQ in 5-year-old children [55], another study conducted in China also finds a negative association between maternal serum Cd and Gesell developmental quotients in 1-year-old children, with brain-derived neurotrophic factor playing a key role [56]. Cd has the capacity to traverse both the placental and blood–brain barriers, facilitating its entry into the fetal circulation [57]. This process can adversely impact the development of the central nervous system through mechanisms such as oxidative stress, dysregulation of hormonal pathways, aberrant expression of genes and their associated proteins, induction of neuronal apoptosis, and disruption of the homeostasis of essential trace elements [58,59]. Furthermore, Sb and its compounds have been recognized as major environmental pollutants in many countries [53]. A study has reported that maternal serum Sb levels are negatively associated with language and social behavior development quotients in 3-year-old children [16]. The neurotoxic effects of Sb could be attributed to neuronal apoptosis, which could result in impaired neurodevelopment [60]. Thus, reducing exposure to both Cd and Sb is essential, especially in vulnerable populations such as pregnant women and young children. Key sources of Cd exposure include diet, smoking, and industrial activities, while Sb exposure in the general population remains low but can rise notably in industrial areas [53,61,62]. In addition to direct neurotoxic effects of Cd and Sb, recent evidence suggests that metabolic perturbations may mediate the impact of prenatal metal exposure on neurodevelopment. It has reported that alterations in histidine, beta-alanine, purine, and pyrimidine metabolism significantly mediated the associations between prenatal metal exposure and cognitive outcomes, suggesting that disruptions in neurotransmitter and neuroendocrine pathways may contribute to impaired neurodevelopment [63]. Addressing these exposure pathways through targeted public health interventions and stricter environmental regulations is crucial for safeguarding childhood development. Interestingly, we did not observe significant contribution of prenatal Pb, Hg, or Se exposure and neurodevelopmental outcomes, which contrasts with some prior studies [64]. One possible explanation is that urinary biomarkers primarily reflect recent exposure and may not capture long-term or cumulative levels of these metals. In contrast, blood measurements are generally considered more indicative of chronic exposure, particularly for Pb and Hg, which may partly explain the discrepancy. To better capture the cumulative burden of toxic metals, future studies should include long-term exposure assessments. Specifically for Hg, hair samples are considered a more reliable biomarker of methylmercury exposure than blood or urine, as they better represent chronic exposure over time. In contrast, urinary mercury mainly reflects inorganic mercury exposure and short-term variations, which may limit its utility in assessing methylmercury-related neurodevelopmental effects. Se, beneficial effects might depend on long-term status rather than short-term fluctuations captured in urine [65].

This study has several strengths. First, the prospective design allows for adjustment of various factors that could affect the results, such as socioeconomic status, lifestyle, and diet. This helps to more accurately assess the relationship between prenatal metal exposure and neurodevelopment in children. Second, using two different indices in the WQS analysis provides a clearer picture of how essential metals and toxic metals differently impact neurodevelopment, as they may work through different biological mechanisms. However, there are also some limitations. First, the relatively small sample size may limit the statistical power of the study, meaning that the results could be less precise and there might be a higher chance of missing an effect. A larger sample size in future research would help strengthen the findings. Second, while urine is commonly used to measure metal exposure, it might not be the best biomarker for all metals, as some metals may be excreted differently. Third, there is a lack of consideration for genetic factors that may influence neurodevelopment, which should be addressed in the future study. Finally, while this study shows an association between prenatal metal exposure and neurodevelopment, the exact mechanisms behind this relationship are not fully understood. More research is needed to clarify how metal exposure affects brain development and whether other factors play a role. Fourth, only a single maternal urine sample was collected during early pregnancy, which may not fully represent exposure throughout gestation. Urinary measurements reflect recent intake and excretion, whereas blood levels may better capture chronic exposure. Future studies using repeated measures or multiple biomatrices could provide a more accurate assessment of prenatal metal exposure. Last, urinary metal concentrations were adjusted for creatinine to account for urine dilution. However, creatinine excretion may vary during pregnancy due to physiological changes, which could introduce variability in the normalized metal concentrations.

In conclusion, prenatal exposure to toxic metals is associated with negative impacts on cognitive and neuropsychological functioning in children. Our findings suggest that pregnant women should be particularly mindful of metal exposure, especially to Cd and Sb, to protect the health and development of their offspring. These results emphasize the importance of public health initiatives aimed at reducing toxic metal exposure during pregnancy. Further research is needed to deepen our understanding of the mechanisms behind these associations and inform policies that can help mitigate potential risks to maternal and child health.

## Figures and Tables

**Table 1:** General characteristics of mother and offspring: sociodemographic data, lifestyle, diet, and neurodevelopment characteristics (*n* = 201).

Maternal Characteristics	Summary Statistics
Age (years), mean ± SD	31.58 ± 4.72
BMI, mean ± SD	24.68 ± 4.27
Social class, *n* (%)	
Low/Medium	150 (74.6)
High	51 (25.4)
Smoking status, *n* (%)	
Never smoker	136 (67.7)
Smoker or ex-smoker	65 (32.3)
MedDiet during pregnancy (score), mean ± SD	9.65 ± 2.44
Children characteristics	
Gestational age (weeks), mean ± SD	39.78 ± 1.37
Sex, *n* (%)	
Male	107 (53.2)
Female	94 (46.8)
Type of feeding, *n* (%)	
Breastfeeding	155 (77.1)
Mixed feeding/infant formula	46 (22.9)
Neurodevelopment of children	
WPPSI score	
VCI	105.27 ± 13.63
FRI	102.67 ± 12.81
WMI	97.75 ± 12.18
PSI	96.21 ± 12.20
FSIQ	102.59 ± 11.28
VAI	97.45 ± 13.92
NVI	101.27 ± 11.54
GAI	106.16 ± 11.85
NEPSY score	
Verbal fluency (language domain)	9.02 ± 2.88
Visual-motor precision (sensorimotor domain)	10.32 ± 3.28
Emotion recognition (social perception domain)	9.01 ± 2.50

Abbreviations: BMI, early pregnancy Body Mass Index; MedDiet, Mediterranean diet; VCI, Verbal Comprehension Index; FRI, Fluid Reasoning Index; WMI, Working Memory Index; PSI, Processing Speed Index; FSIQ, Full Scale IQ; VAI, Vocabulary Acquisition Index; NVI, Nonverbal Index; GAI, General Ability index; WPPSI, Wechsler Preschool and Primary Scale of Intelligence; NEPSY, Developmental Neuropsychological Assessment;.

**Table 2:** Urinary concentrations of metals in pregnant women.

		Percentile		GM	IQR
	25th	50th	75th		
Adjusted (μg/g of Creatinine)					
Essential metals					
Mg	5.49 (×10^4^)	7.32 (×10^4^)	9.86 (×10^4^)	7.43 (×10^4^)	4.27 (×10^4^)
Cr	0.13	0.23	0.35	0.26	0.21
Mn	0.00	0.02	0.09	0.11	0.23
Mo	29.34	40.98	55.98	39.87	26.78
Co	0.18	0.29	0.58	0.35	0.39
Cu	5.08	7.23	10.00	6.71	4.88
Zn	198.02	304.19	430.90	271.80	233.81
Se	21.62	26.12	31.03	26.67	8.80
Toxic metals					
As	9.84	24.21	61.37	30.44	67.62
Cd	0.14	0.23	0.33	0.22	0.19
Sb	0.03	0.05	0.08	0.05	0.04
Hg	0.29	0.57	0.94	0.50	0.63
Pb	0.16	0.36	0.68	0.37	0.45
Ni	1.08	1.59	2.38	1.70	1.33

Abbreviations: Mg, Magnesium; Cr, Chromium; Mn, Manganese; Mo, Molybdenum; Co, Cobalt; Cu, Copper; Zn, Zinc; Se, Selenium; As, Arsenic; Cd, Cadmium; Sb, Antimony; Hg, Mercury; Pb, Lead; Ni, Nickel; GM, Geometric Mean; IQR, Interquartile Range.

**Table 3:** The joint effect of essential and toxic metals on neurodevelopment by WPPSY score using WQS.

	VCI		FRI		WMI		PSI		FSIQ		VAI		NVI		GAI	
	est	95%CI	est	95%CI	est	95%CI	est	95%CI	est	95%CI	est	95%CI	est	95%CI	est	95%CI
β WQS (Essential metals)	0.69	−5.36, 6.22	−1.05	−2.30, 0.15	4.79	−1.66, 10.45	−1.02	−2.17, 0.04	1.90	−2.22, 6.87	0.80	−0.49, 2.20	−1.00	−2.21, 0.07	0.32	−4.63, 6.45
β WQS^2^ (Essential metals)	−0.12	−0.82, 0.66			−0.59	−1.75, 0.16			−0.38	−1.10, 0.30					−0.17	−1.16, 0.60
β WQS (Toxic metals)	**−1.07**	**−2.03, −0.10**	0.28	−0.70, 1.23	0.50	−0.38, 1.35	**−0.98**	**−1.73, −0.20**	−0.28	−1.18, 0.42	**−1.25**	**−2.18, −0.23**	−0.08	−1.01, 0.79	−0.34	−1.48, 0.27

Abbreviations: est. Estimate; WPPSY, Wechsler Preschool and Primary Scale of Intelligence; WQS, Weighted Quantile Sum; WQS^2^, Quadratic term for WQS; CI, confidence interval; VCI, Verbal Comprehension Index; FRI, Fluid Reasoning Index; WMI, Working Memory Index; PSI, Processing Speed Index; FSIQ, Full Scale IQ; VAI, Vocabulary Acquisition Index; NVI, Nonverbal Index; GAI, General Ability index. The value in bold indicates statistical significance.

**Table 4:** The joint effect of essential and toxic metals on neurodevelopment by NEPSY score using WQS.

	Verbal Fluency (Language Domain)		Visual-Motor Precision (Sensorimotor Domain)		Emotion Recognition (Social Perception Domain)	
	est	95%CI	est	95%CI	est	95%CI
β WQS (Essential metals)	−0.07	−0.30, 0.21	−0.15	−0.35, 0.28	0.15	−0.03, 0.34
β WQS (Toxic metals)	**−0.23**	**−0.38, −0.03**	0.17	−0.04, 0.50	0.09	−0.08, 0.26

Abbreviations: est. Estimate. NEPSY, Developmental Neuropsychological Assessment; WQS, Weighted Quantile Sum; CI, confidence interval; The value in bold indicates statistical significance.

## Data Availability

The data presented in this study are available on request from the corresponding author.

## Supplementary Materials

The following supporting information can be downloaded at: https://www.mdpi.com/article/10.3390/toxics13110954/s1, Table S1: Effective degrees of freedom and significance of smooth terms in metals and neurodevelopment by WPPSI score using GAM; Table S2: Effective degrees of freedom and significance of smooth terms in metals and neurodevelopment by NEPSY score using GAM title.
