# Supplementary material for: Effects of Prenatal Essential and Toxic Metal Exposure on Children’s Neurodevelopment: A Multi-Method Approach
Source: Toxics. 2025 Nov 5;13(11):954. doi: 10.3390/toxics13110954 (PMC12656216; doi:10.3390/toxics13110954)
Supplement: Supplementary file 1 [file toxics-13-00954-s001.zip › Supplementary Tables.pdf]

Supplementary Table S1. Effective degrees of freedom and significance of smooth terms in metals and neurodevelopment by WPPSI score using GAM

	VCI	FRI	WMI	PSI	FSIQ	VAI	NVI	GAI
Essential metals	edf	edf	edf	edf	edf	edf	edf	edf
Mg	1.00	1.00	**1.00**	1.00	1.02	1.00	1.00	1.26
Cr	1.00	1.00	1.64	1.00	1.00	1.00	1.00	1.00
Mn	**1.81**	1.75	**1.87**	1.00	1.49	1.46	1.23	**1.70**
Mo	**1.90**	1.28	1.14	1.44	**1.87**	1.62	1.32	**1.91**
Co	1.00	1.00	1.00	1.00	1.49	1.00	1.00	1.15
Cu	1.00	1.00	1.67	1.00	1.00	1.00	1.00	1.00
Zn	1.54	**1.00**	1.00	1.00	1.00	1.00	1.00	**1.00**
Se	1.51	1.00	1.00	1.00	1.53	1.00	1.24	1.45
Toxic metals								
As	1.21	1.00	1.31	1.31	1.10	1.00	1.00	1.46
Cd	**1.00**	1.00	1.00	1.00	1.00	**1.00**	1.00	**1.00**
Sb	1.00	1.68	1.00	**1.00**	1.00	1.00	1.00	1.00
Hg	1.00	1.59	1.00	1.00	1.00	1.45	1.23	1.00
Pb	1.00	1.00	1.00	1.00	1.00	1.00	1.00	1.00
Ni	1.00	**1.00**	1.00	1.68	1.00	1.00	1.00	1.00

Abbreviations: WPPSI, Wechsler Preschool and Primary Scale of Intelligence; GAM, Generalized Additive Model; Mg, Magnesium; Cr, Chromium; Mn, Manganese; Mo, Molybdenum; Co, Cobalt; Cu, Copper; Zn, Zinc; Se, Selenium; As, Arsenic; Cd, Cadmium; Sb, Antimony; Hg, Mercury; Pb, Lead; Ni, Nickel;VCI, Verbal Comprehension Index; FRI, Fluid Reasoning Index; WMI, Working Memory Index; PSI, Processing Speed Index; FSIQ, Full Scale IQ; VAI, Vocabulary Acquisition Index; NVI, Nonverbal Index; GAI, General Ability index; edf, effective degrees of freedom. The value in bold indicates statistical significance.

Supplementary Table S2. Effective degrees of freedom and significance of smooth terms in metals and neurodevelopment by NEPSY score using GAM

	Verbal fluency (language domain)	Visual-motor precision (sensorimotor domain)	Emotion recognition (social perception domain)
	edf	edf	edf
Essential metals			
Mg	1.59	1.00	1.00
Cr	1.51	1.00	1.45
Mn	1.00	1.00	1.47
Mo	1.00	1.74	1.20
Co	1.55	1.30	1.00
Cu	1.07	1.00	1.00
Zn	**1.00**	1.00	1.00
Se	1.09	1.00	1.69
Toxic metals			
As	1.00	1.00	1.49
Cd	**1.00**	1.00	1.49
Sb	1.00	1.42	1.00
Hg	1.44	1.44	1.55
Pb	1.00	1.00	1.00
Ni	1.00	1.32	1.46

Abbreviations: NEPSY, Developmental Neuropsychological Assessment; GAM, Generalized Additive Model; Mg, Magnesium; Cr, Chromium; Mn, Manganese; Mo, Molybdenum; Co, Cobalt; Cu, Copper; Zn, Zinc; Se, Selenium; As, Arsenic; Cd, Cadmium; Sb, Antimony; Hg, Mercury; Pb, Lead; Ni, Nickel; edf, effective degrees of freedom.The value in bold indicates statistical significance.
